# Metagenomic analysis of the effects of salinity on microbial community and functional gene diversity in glacial meltwater estuary, Ny-Alesund, Arctic

**DOI:** 10.1007/s42770-024-01298-x

**Published:** 2024-04-22

**Authors:** Fan Yang, Qinxin Li, Xiaofei Yin

**Affiliations:** 1 Management College, Ocean University of China, Qingdao, China; 2 Business College, Qingdao University, Qingdao, China; 3 College of Chemistry and Chemical Engineering, Qingdao University, Qingdao, China; 4 First Institute of Oceanography, Ministry of Natural Resources, Qingdao, China

**Keywords:** Metagenome, Marine microorganism, Bacterial diversity, Nitrogen cycle, Sulfur cycle

## Abstract

Due to the inflow of meltwater from the Midre Lovénbreen glacier upstream of Kongsfjorden, the nutrient concentration of Kongsfjorden change from the estuary to the interior of the fjord. Our objective was to explore the changes in bacterial community structure and metabolism-related genes from the estuary to fjord by metagenomic analysis. Our data indicate that glacial meltwater input has altered the physicochemical properties of the fjords, with a significant effect, in particular, on fjords salinity, thus altering the relative abundance of some specific bacterial groups. In addition, we suggest that the salinity of a fjord is an important factor affecting the abundance of genes associated with the nitrogen and sulfur cycles in the fjord. Changes in salinity may affect the relative abundance of microbial populations that carry metabolic genes, thus affecting the relative abundance of genes associated with the nitrogen and sulfur cycles.

**Supplementary Information:**

The online version contains supplementary material available at 10.1007/s42770-024-01298-x.

## Introduction

Over the past few decades, the global climate has changed dramatically, with the effects becoming more pronounced in the polar regions [1]. This would lead to higher temperatures in the Arctic Ocean, including Spitsbergen, causing the glaciers to melt earlier in the annual cycle and then freeze, increasing precipitation and reducing sea ice cover. In addition, glacial melt drains downstream, leading to changes of biogeochemical and nutrient salt in downstream ecosystems [2–7]. Due to the strong changes in salinity and chemical properties of seawater, the structure and abundance of the microbial community in downstream fjords and estuaries will change. Microorganisms in marine ecosystems are the most important drivers of biogeochemical cycling on a global scale [8], as they are responsible for the remineralization of organic matter and the transfer of nutrients and energy to higher levels in the ocean [9, 10]. However their taxonomic composition and metabolic function are influenced by "bottom-up" physical and chemical factors, such as salinity, nutrient concentration, and availability [11, 12]. Therefore, the natural processes that cause physiochemical dynamics changes greatly affect the bacterial community, resulting in rapid changes in community structure and function, and thus have an impact on the aquatic environment [13].

Kongsfjorden, located on the west coast of Svalbard at 79°N, is a glacier-open fjord in the Arctic. Since arctic glacial fjords are characterized by the discharge of fresh water and suspended matter from glaciers on top of fjords, the stable ocean at the entrance to the fjord becomes a very unstable brackish water in the inner basin of the fjord [14]. Because the Arctic was significantly affected by global warming, and this process leads to significant changes in the nearshore ecosystem, Kongsfjorden has attracted extensive research attention in recent years. Previous studies have shown that changes in salinity and sediment load due to the inflow of glacial meltwater into Kongsfjorden are the main determinants of changes in microbial community composition and diversity in Kongsfjorden [15], consistent with another research that indicated salinity is a selective pressure governing global bacterial distribution [11]. However, the characteristics and metabolic potential of the fjord bacterial community have not been determined. Since bacteria are the basis of major biogeochemical cycles, the changes in high trophic levels and the whole ecosystem will determine the community’s characteristics and functions. Therefore, how changes in nutrient concentrations affect bacterial communities is important. Metagenomics overcomes the constraints faced by culture-oriented microbiology institutions and can be used as a search tool for detailed screening of microbial community species present in ecosystems [16]. Because DNA extracted from environmental samples is a microcosm of the entire microbial community of an ecosystem, metagenomic analysis can provide a more comprehensive assessment of the entire microbial community [17]. Thus, advances in community analysis using metagenomic methods, have made it possible to characterize the bacterial taxonomic composition and functional metabolic potential. In this case, the purpose of this study was to investigate the changes in bacterial community structure and the relative abundance of metabolic genes from the glacial meltwater estuary to the interior of Kongsfjorden. To do this, we set up three sampling sites in Kongsfjorden: the intersection of glacial meltwater and the edge of the fjord (S5), the coastal waters (S6), and the interior of Kongsfjorden (S7). The nutrient concentration of the three sites varies significantly due to the input of glacial meltwater and their location in the fjords. We expect changes in bacterial community structure and abundance of major metabolic genes from the fjord edge to the fjord interior.

## Materials and Methods

### Study sites and sample collection

Kongsfjorden is a polar fjord located west of Svalbard located between 78°04′N-79°05′N and 11°03′E-13°03′E. The fjord is characterized by a low tidal difference (~ 2 m) and is strongly influenced by topography and the adjacent ocean. The western coastal waters of Svalbard are affected by the northernmost extension of the North Atlantic Current. The Midre Lovénbreen glacier is located in the Kongsfjorden region. Glacial meltwater flows into the Kongsfjorden through glacial runoff and provides the main freshwater resources. So nutrient concentrations will change from the inlet of glacial meltwater to the interior of the fjord [15].

**Fig.1:**
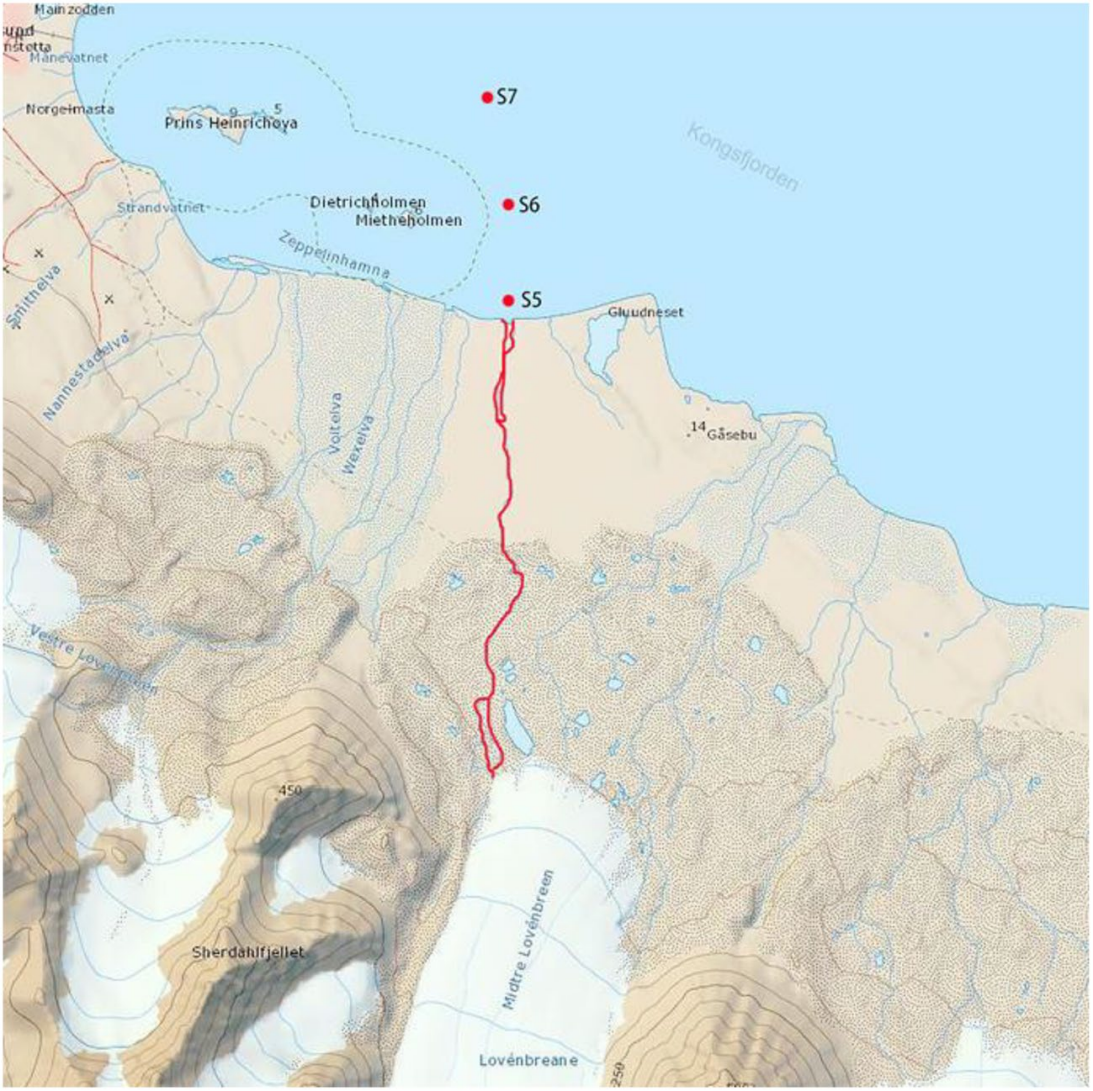
Sampling sites in the Kongsfjorden

As shown in Fig. 1 (https://toposvalbard.npolar.no/), three sampling points will be set up in the waters of Kongsfjorden, namely a place where the estuary of Kongsfjorden (S5, 12°01´50.9"E and 78°54´54.2"N), the coastal sea area (S6, 12°02´6.9"E and 78°54´55.7"N), and interior of the fjord (S7, 12°02´32.8"E and 78°55´2.7"N), and the sampling points are 500 m apart. Three parallel superficial water samples were taken from each sampling point, and water samples were collected directly into TWIRL’EM sterile sampling bags (Labplas Inc., Canada). The microbial samples were then collected by filtering 1000 ml of the water samples. The microbial biomass was successively trapped onto 47-mm-diameter, 0.2-μmpore-size membrane filters (Pall Corporation, USA). Membrane filters were placed in centrifuge tubes at − 20 °C in the Yellow River Station (China) and taken to the laboratory by plane. Filters were then frozen at − 80 °C until nucleic acid extraction.

### The chemical properties of seawater

Temperature and salinity were measured by a CTD (SBE 19 plus CTD, Sea-Bird Electronics, Bellevue, Washington, USA) at each sampling site. The pH of seawater was measured by pH electrode (PHS-3C, Shanghai REX Instrument Factory, Shanghai, China). NO_3_^−^-N, NO_2_^−^-N, NH_4_^+^-N, SiO_3_^2−^-Si, and PO_4_^3−^-P concentration of seawater samples were measured by nutrient automatic analyzer (QuAAtro, SEAL, Germany), the relative standard deviation of physical and chemical properties < 5%.

### Metagenomic sequencing and analyses

The total DNA was extracted from the water sample using the PowerWater DNA Isolation Kit (MO BIO Laboratories, Inc., USA) according to the manufacturer's instructions. A total of 1.0 μg DNA per sample was used for library construction with a NEBNext Ultra DNA Library Prep Kit for Illumina (New England Biolabs, Ipswich, MA, USA) following the manufacturer’s recommendations and corresponding index sequences. Briefly, DNA samples were fragmented by sonication to 350 bp, and fragments were end-polished, A-tailed, and ligated with the full-length adaptor for PCR amplification. PCR products were purified (AMPure XP system; Beckman Coulter, Brea, CA, USA), and libraries were analyzed for size distribution by an Agilent 2100 Bioanalyzer and quantified using real-time PCR. The clustering of index-coded samples was performed on a cBot Cluster Generation System according to the manufacturer’s instructions. After cluster generation, library preparations were sequenced on an Illumina HiSeq 2000, and paired-end reads were generated and deposited to NCBI SRA (accession number: SRP319744).

In the metagenome analysis, reads of low quality (containing 40 or more bases with scores < 20) or with more than 10 unknown bases (Ns) were removed using SOAPaligner [18]. Clean reads from each sample were assembled using SOAPdenovo2 using default parameters [19]. The reads not assembled into contigs (continuous sequences within scaffolds) in each sample were pooled together to form a mixed sample, which was reassembled. All contigs shorter than 500 bp were discarded.

Open reading frames in the contigs of each sample and the mixed sample were predicted using MetaGeneMark(V2.10) [20]. Redundant genes in each sample were removed using CD-HIT (4.5.8) with 95% identity and 90% coverage [21]. The resulting genes were further filtered with the number of reads assembled, and those assembled with less than two reads were discarded to generate a gene catalogue (Unigenes). Unigenes were then filtered using DIAMOND (V0.9.9.110) [22] against the reference genes in the NCBI-NR database at a cut-off e-value of 1E-10. In the functional analysis, DIAMOND was used to map Unigenes against reference genes in KEGG [23], and the relative abundance of different functional levels was counted.

### Statistical analysis

The Principal Coordinate Analysis (PCoA) on weighted UniFrac distances was computed in R (V4.0.5) to examine dissimilarities between nine samples in three sites. Correlation and significance analysis, such as one-way analysis of variance (ANOVA), Pearson correlation between genes and chemical properties, were conducted using IBM SPSS Statistics 26.

## Results

### Chemical properties of the samples

Due to the damage of a parallel sample S7.2 from the sampling site S7 during transportation, there are only two parallel samples of S7 in the subsequent experiment. However, from the perspective of various indicators of chemical properties, S7 parallel sample has very good parallelism, so it does not affect the subsequent experimental results.

The salinity of the three sampling sites varied greatly (one-way ANOVA, *P* < 0.05; Table S1), showing an increasing trend from S5 to S7 (Table 1). In addition to salinity, other six chemical factors which are important parameters in marine research were determined at the three sampling sites: pH, NH_4_^+^-N, NO_3_^−^-N, NO_2_^−^-N, PO_4_^3−^-P, and SiO_3_^2−^-Si. The results showed that the maximum values of SiO_3_^2−^-Si (92.48 μg/g) and NO_3_^−^-N (5.26 μg/g) were detected in S7 samples, and the maximum values of NH_4_^+^-N (33.29 μg/g) and NO_2_^−^-N (3.24 μg/g) were detected at S6 sampling points. In general, salinity was the chemical property with the greatest difference among the three sampling sites, so we inferred that salinity may have a certain influence on the driving bacterial community structure and gene diversity.

**Table 1:** Chemical properties of three sampling sites

sample	pH	salinity (ppt)	NH_4_^+^-N (μg/g)	SiO_3_^2−^-Si (μg/g)	NO_2_^−^-N (μg/g)	PO_4_^3−^-P (μg/g)	NO_3_^−^-N (μg/g)
S5.1	8.01	5.08	33.213	91.039	3.151	5.384	5.251
S5.2	8.00	5.16	31.221	90.081	3.230	5.390	5.153
S5.3	8.02	5.06	33.199	89.370	3.222	5.313	5.043
Average	8.01^a^	5.10^c^	32.544^a^	90.163^b^	3.201^a^	5.362^a^	5.149^a^
S6.1	8.06	15.21	32.570	92.410	3.240	5.357	5.247
S6.2	8.02	15.09	32.641	91.550	3.205	5.370	5.248
S6.3	8.04	15.06	33.290	91.510	3.250	5.221	5.246
Average	8.04^a^	15.12^b^	32.834^a^	91.823^a^	3.232^a^	5.316^a^	5.247^a^
S7.1	7.95	30.10	32.195	92.480	3.051	5.334	5.251
S7.2	8.00	30.12	32.573	90.653	3.191	5.280	5.260
S7.3	7.93	30.11	32.413	91.120	3.153	5.070	5.257
Average	7.96^b^	30.11^a^	32.394^a^	91.418^a^	3.132^a^	5.228^a^	5.256^a^

Statistical significance was assessed by one-way ANOVA followed by Tukey’s HSD test, and significant differences were accepted when *p* < 0.05 between the two groups. The letters a, b, and c were used to show statistically significant differences

### Metagenomic sequencing results and gene prediction

**Fig.2:**
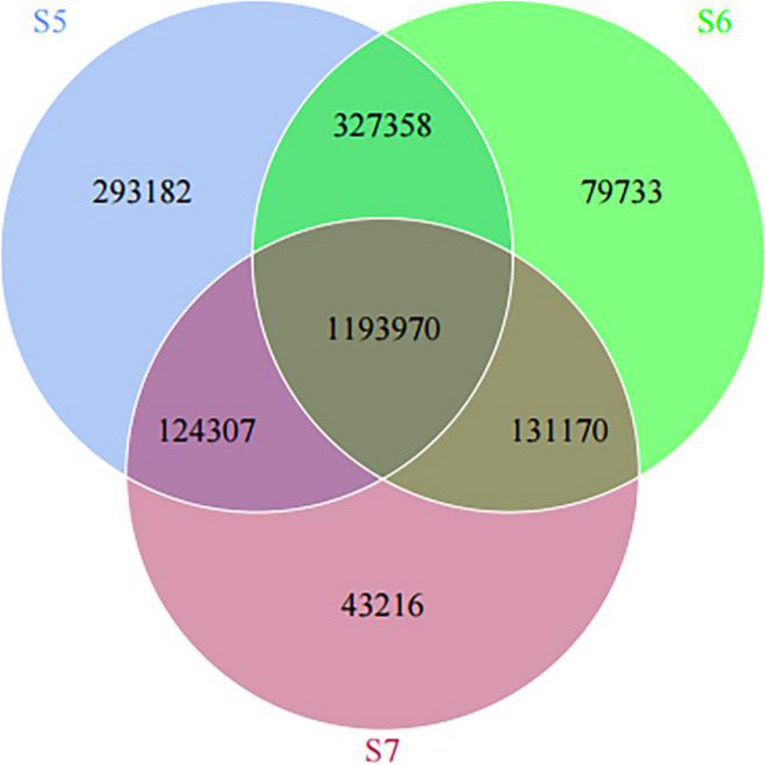
A Venn diagram showing the degree of gene overlap between the three sample sites

Illumina HiSeq sequencing platform was used to obtain the original data, and the following data were obtained after preprocessing and statistics (Table 2). A total of 111,529.18 Mbp sequences were obtained from all the samples, and the average sequencing number of each sample reached 13,941.14 Mbp. After quality control, the remaining 111,402.52 Mbp sequences averaged 13,925.315 Mbp per sample, with an average effective rate of 99.89%. The average percentage of the number of bases with sequencing error rate less than 0.01 (mass value greater than 20) in CleanData was 97.03, indicating a low error rate in the sample. The average GC content of base in CleanData was 44.26%. We conducted gene count statistics for each sampling site and drew a Venn diagram (Fig. 2), among which 1,193,970 genes were shared by the three sampling sites. The number of unique genes for S5, S6, and S7 was 293,182, 79,733, and 43,216, respectively.

**Table 2:** Data preprocessing statistics

Sample	RawData (Mbp)	CleanData (Mbp)	Clean_Q20 (%)	Clean_Q30 (%)	Clean_GC (%)	Effective (%)
S5.1	16,505.17	16,490.68	97.72	93.53	43.57	99.912
S5.2	13,521.45	13,499.78	97.68	93.35	43.75	99.840
S5.3	14,532.84	14,523.57	96.60	90.95	43.12	99.936
S6.1	13,843.90	13,825.51	96.39	90.66	45.17	99.867
S6.2	11,985.79	11,975.3	96.84	91.55	44.78	99.913
S6.3	14,247.90	14,224.63	96.77	91.56	45.91	99.837
S7.1	14,763.39	14,747.21	97.30	92.50	44.07	99.890
S7.3	12,128.74	12,115.84	96.97	91.82	43.7	99.894

Unigenes were sequenced and annotated with NCBI NR database for bacteria, fungi, archaea, and virus respectively. It was found that the microorganisms in the samples were mainly bacteria, accounting for 84% (S5), 77.1% (S6) and 82.3% (S7), Viruses for 3.8% (S5), 5% (S6) and 4% (S7), Eukarya for 0.3% (S5), 0.7% (S6) and 0.5% (S7) and archaea for 0.2% (S5), 0.2% (S6) and 0.2% (S7). In the metagenomic library of 3 sample sites, 11.7%, 17%, and 13% of the genes were unannotated, respectively.

### Changes in bacterial community composition

**Fig.3:**
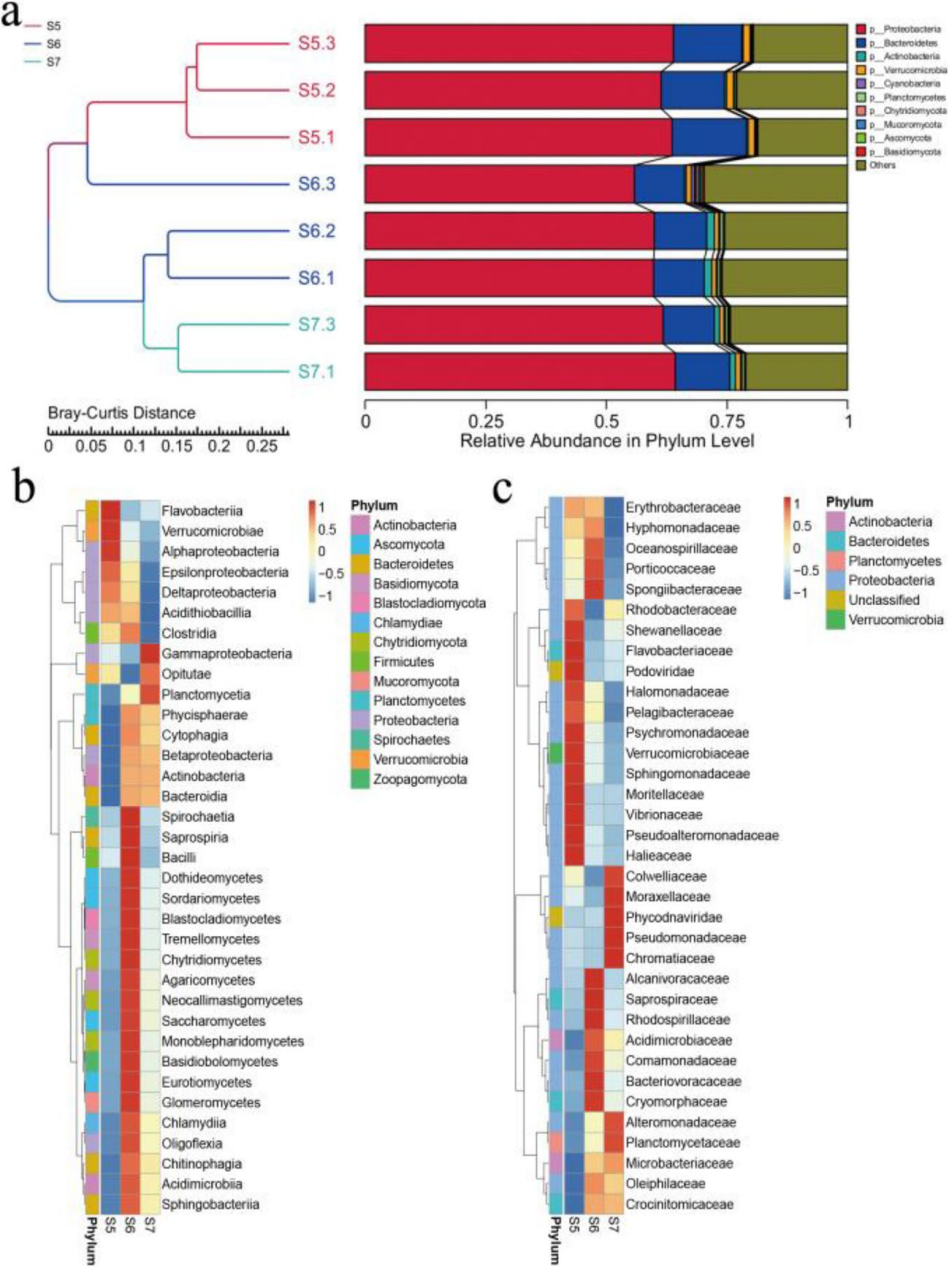
Microbial UPGMA clusters and relative abundance of species at the phyla level (a); A heatmap of the top 35 abundant classes (b); A heatmap of the top 35 abundant families (c)

Proteobacteria were abundant in all three sampling sites at 58.51%-62.98% (Fig. 3a). Alphaproteobacteria (25.79%) and Deltaproteobacteria (0.35%) were relatively abundant in S5, while the abundance of Gammaproteobacteria (39.67%) and Betaproteobacteria (1.5%) was higher in S7 (Fig. 3b, one-way ANOVA, *P* < 0.05; Table S2). At the family level, the abundance of Rhodobacteraceae (12.23%), Pseudoalteromonadaceae (8.45%), Flavobacteriaceae (10.44%), and Vibrionaceae (0.69%) was relatively high in S5 (Fig. 3c, one-way ANOVA, *P* < 0.05; Table S3). As the sampling sites got deeper into the fjord, the abundance of Actinobacteria (0.38%-1.2%) increased gradually. Especially, the abundance of Acidimicrobiaceae (0.06%-0.26%) and Microbacteriaceae (0.08%-0.68%), which belong to Actinobacteria, was higher in S6 and S7 than in S5. At the family level, the abundance of Colwelliaceae (8.33%), Chromatiaceae (0.46%), and Alteromonadaceae (11.42%) were higher in S7 than that in S5 (one-way ANOVA, *P* < 0.05; Table S4).

**Fig.4:**
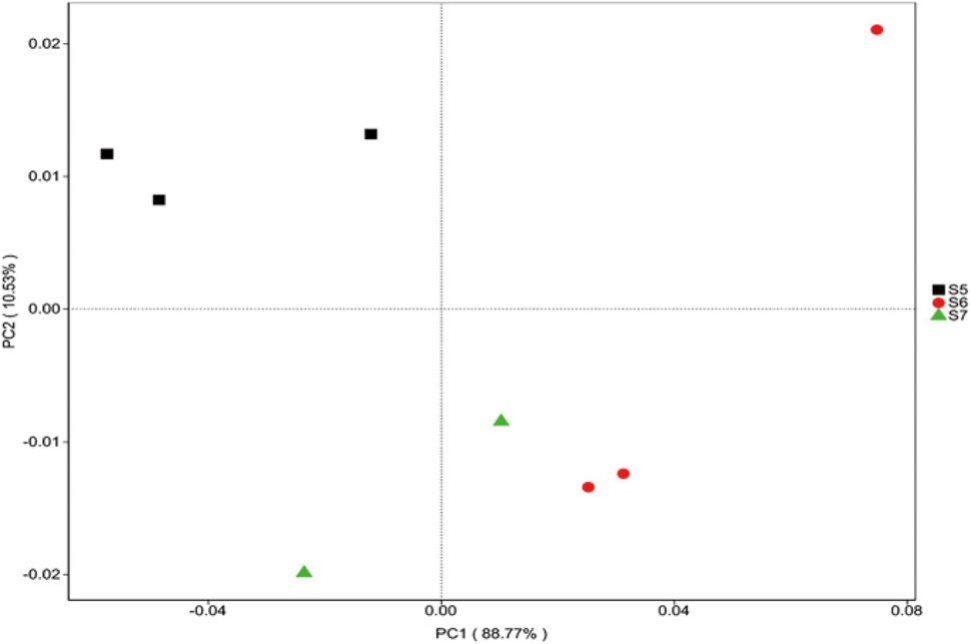
Principal coordinate analysis based on weighted UniFrac distances

Dissimilarities between samples using PCoA were examined based on weighted UniFrac distances (Fig. 4). As expected, the three groups of samples were significantly separated, and the S5 sample was separated from the other two groups, distributed along the first principal component. The distribution pattern of S6 sample was distributed along the second principal component, which was opposite to S5.

### Changes in metabolic function genes

Starting from the results of Unigenes annotation, the KEGG database was selected for annotation. A total of 780,765 genes were annotated, and a total of 45 pathways were obtained. As shown in the figure (Fig. S1), the abscissa on the bar chart shows the number of Unigenes on the notes, and the ordinate with different colors represents the six metabolic pathways in the KEGG database, including Cellular Processes, Environmental Information Processing, Genetic Information Processing, Human Diseases, Metabolism and Organismal Systems. Different columns represent different pathways of the six metabolic fluxes, and the corresponding explanations are shown in the legend on the left. In the samples, the main genes were distributed in Amino acid metabolism (96,066 Unigenes), Carbohydrate metabolism (86,517 Unigenes), Metabolism of cofactors and vitamins (60,104 Unigenes), and Energy metabolism (56,892 Unigenes).

**Fig.5:**
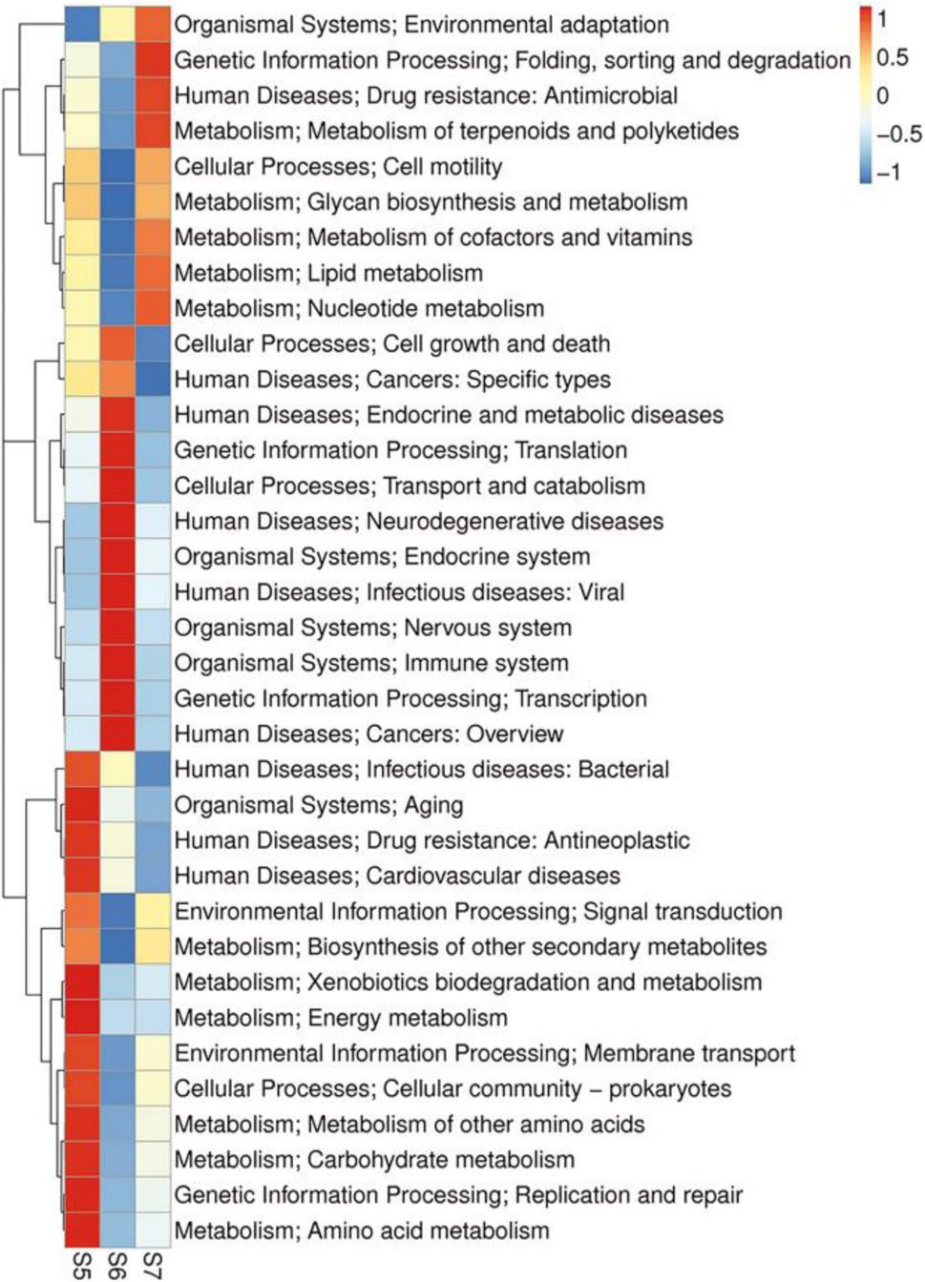
The top 35 functional genes with the highest gene abundance

By distributing the top 35 functional genes with the highest gene abundance (Fig. 5), it can be found that there were great differences in gene functional abundance among the three sampling points, especially S5 and S7. We focused on the abundance of metabolism-related genes and found that most metabolism-related genes were abundant in S5, especially in Energy metabolism (3.09%), Amino acid metabolism (5.05%), and Carbohydrate metabolism (4.31%), while Nucleotide metabolism (2.39%) and Lipid metabolism (1.33%) were abundant in S7.

### Changes in genes associated with the nitrogen cycle

**Fig.6:**
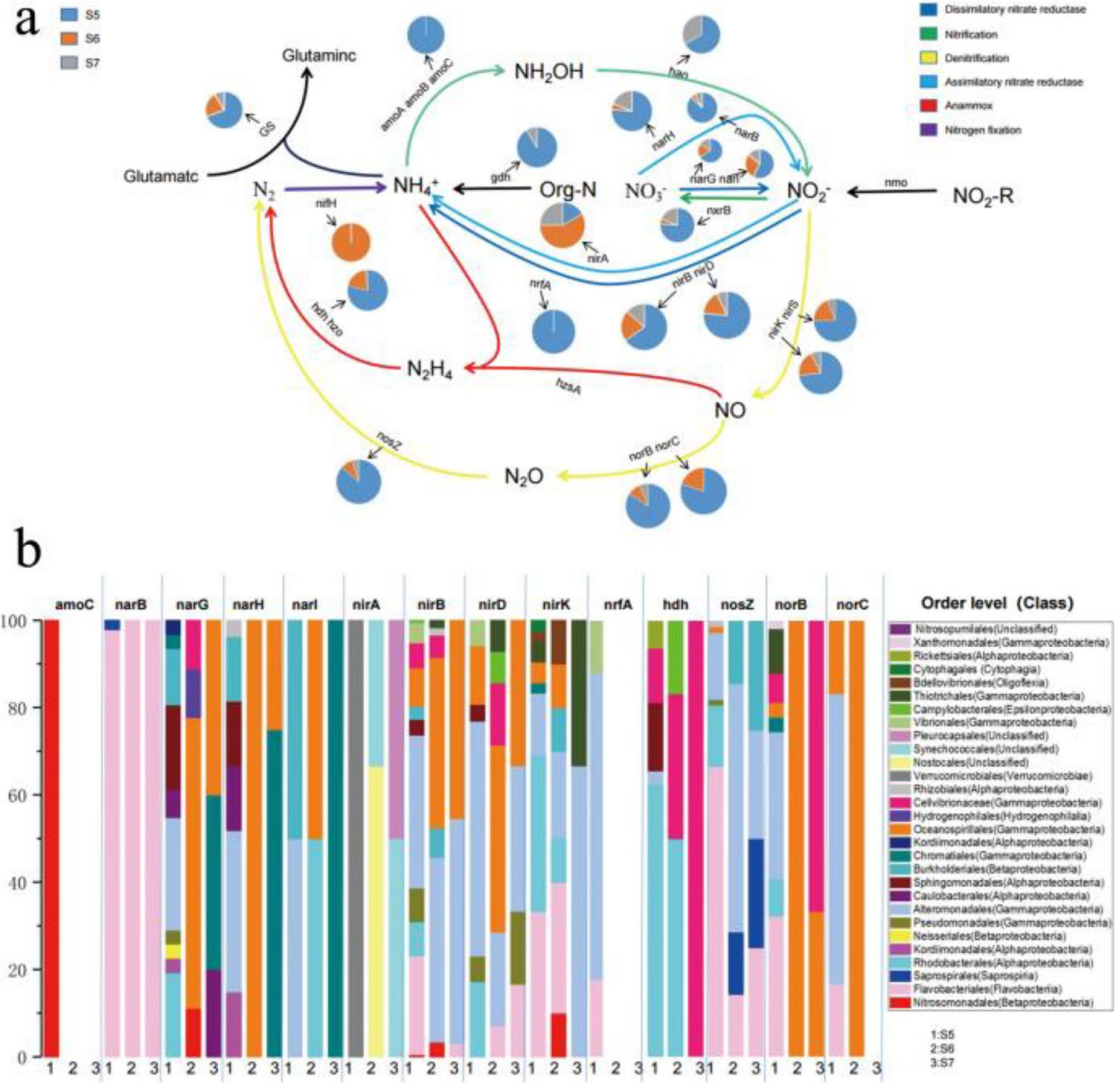
The pie charts represent the relative abundance of nitrogen metabolism- related genes, which was calculated through dividing the sum of a gene’ s coverage in an individual sample by the sum of this gene’ s coverage in all samples (a); Microbial taxa of nitrogen cycling-related genes and their relative abundance in each sample (b)

In the KEGG annotated gene, a total of six nitrogen metabolism patterns and related genes were detected (Fig. 6a), including nitrification (*amoC*, *nxrB*, and *hao*), denitrification (*nirS*, *nirK*, *norC*, and *norB*), dissimilatory nitrate reductase (*narG*, *narI*, *nirD*, *nirB* and *nrfA*), assimilatory nitrate reductase (*narH*, *narB*, and *nirA*), anammox (*hdh*) and nitrogen fixation (*nifH*). Two exogenous ammonia input-related genes, glutamine synthetase (*GS*) and glutamate dehydrogenase (*gdh***)** were widely distributed throughout the sampling site. Ammonia Monooxygenase Subunit C (*amoC*) was only detected in S5. Denitrification-related genes (*nirK*, *nirS*, *norC*, *norB*, and *nosZ*) and Anammox-related genes (*hdh*) were rarely distributed in S7. In addition, in terms of abundance, the abundance of most genes related to the nitrogen cycle in S5 was higher than that in the other two sample sites. We analyzed the correlation between the abundance and chemical properties of nitrogen cycling-related genes at each sampling site (Table 3). Some genes of dissimilated nitrate reductase and assimilative nitrate reductase were not highly correlated with salinity, while most of the genes related to the nitrogen cycle were highly correlated with salinity.

**Table 3:** Pearson correlation between nitrogen cycling-related genes and chemical properties

		pH	salinity	NH_4_^+^-N	SiO_3_^2−^-Si	NO_2_^−^-N	PO_4_^3−^-P	NO_3_^−^-N
*amoC*	*r*	-0.002	-0.749*	0.165	-0.843**	0.112	0.281	0.331
	*P*	0.996	0.033	0.697	0.009	0.792	0.5	0.423
*narB*	*r*	-0.138	-0.753*	0.014	-0.703	-0.047	0.461	0.005
	*P*	0.745	0.031	0.973	0.052	0.912	0.25	0.991
*narG*	*r*	-0.117	-0.252	-0.54	0.2	-0.113	0.648	-0.840**
	*P*	0.783	0.547	0.167	0.636	0.789	0.082	0.009
*narH*	*r*	-0.169	-0.194	-0.148	-0.081	-0.02	-0.087	-0.605
	*P*	0.69	0.645	0.726	0.848	0.963	0.837	0.112
*narI*	*r*	0.138	-0.379	-0.231	0.287	0.071	0.484	-0.732*
	*P*	0.745	0.355	0.582	0.491	0.867	0.225	0.039
*nirA*	*r*	0.284	0.24	0.383	0.327	0.278	-0.850**	-0.008
	*P*	0.496	0.567	0.349	0.429	0.506	0.008	0.986
*nirB*	*r*	0.23	-0.806*	0.219	-0.516	0.156	0.366	0.218
	*P*	0.584	0.016	0.602	0.19	0.713	0.373	0.603
*nirD*	*r*	-0.026	-0.664	-0.038	-0.303	-0.084	0.368	-0.089
	*P*	0.95	0.072	0.929	0.465	0.843	0.369	0.834
*nirK*	*r*	-0.005	-0.871**	-0.027	-0.765*	0.132	0.516	0.044
	*P*	0.991	0.005	0.949	0.027	0.755	0.191	0.919
*nrfA*	*r*	-0.053	-0.732*	0.134	-0.562	-0.046	0.465	0.027
	*P*	0.902	0.039	0.751	0.147	0.914	0.246	0.949
*hdh*	*r*	0.03	-0.725*	-0.09	-0.268	-0.09	0.806*	-0.049
	*P*	0.944	0.042	0.832	0.521	0.832	0.016	0.909
*nosZ*	*r*	-0.029	-0.830*	-0.152	-0.791*	0.157	0.544	-0.045
	*P*	0.946	0.011	0.72	0.019	0.71	0.164	0.916
*norB*	*r*	0.329	-0.893**	0.143	-0.666	0.447	0.34	0.078
	*P*	0.426	0.003	0.736	0.071	0.267	0.41	0.855
*norC*	*r*	0.252	-0.935**	0.003	-0.635	0.329	0.566	0.045
	*P*	0.547	0.001	0.994	0.091	0.427	0.144	0.915

*Correlation is significant at the 0.05 level; **Correlation is significant at the 0.01 level

To distinguish the microbial groups to which the genes related to nitrogen metabolism belong, the relative abundance map of the groups to which the genes belong was drawn (Fig. 6b). The results showed that different nitrogen cycling-related genes belonged to different microbial groups. For example, the denitrification related gene *nosZ* related microbial taxa in S5 were mainly composed of Gammaproteobacteria, Alphaproteobacteria and Flavobacteria, while the denitrification related gene *nosZ* related microbiota in S6 and S7 were mainly composed of Gammaproteobacteria. However, in general, the microbial taxa belonging to nitrogen cycling related genes at low salinity sites were mainly composed of Gammaproteobacteria and Alphaproteobacteria, while those at high salinity sites were mainly composed of Gammaproteobacteria.

### Changes in genes associated with the sulfur cycle

**Fig.7:**
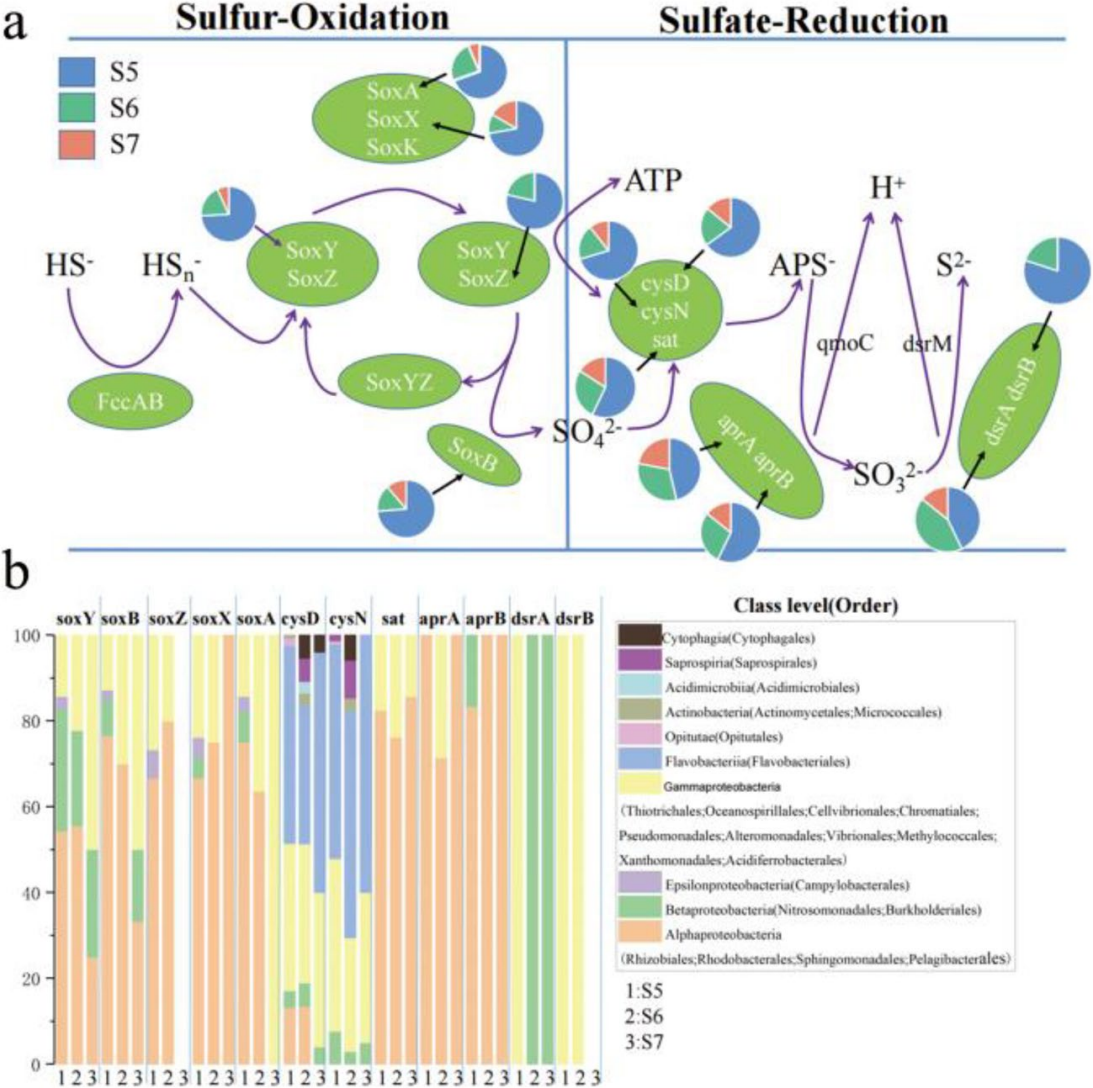
The pie charts represent the relative abundance of sulfur metabolism-related genes, which was calculated by dividing the sum of a gene's coverage in an individual sample by the sum of this gene's coverage in all samples (a); Microbial taxa of sulfur cycling-related genes and their relative abundance in each sample (b)

In our KEGG annotated gene, the sulfur cycle was mainly controlled by two metabolic processes, namely sulfur oxidation and sulfur reduction [24] (Fig. 7a). Our results indicated that sulfur cycling-related genes accounted for the largest proportion in S5. The sulfur oxidation genes (*SoxA*, *SoxX*, *SoxY*, *SoxZ* and *SoxB*) were similar in composition to related microorganisms (Fig. 7b), mainly carried by Alphaproteobacteria, Betaproteobacteria, and Gammaproteobacteria. The *cysD* and *cysN* genes showed similar microbial group distribution among the three sampling sites, which were mainly carried by Flavobacteriia, and Gammaproteobacteria. In addition, in S5, both *dsrA* and *dsrB* genes were carried only by the Gammaproteobacteria, while in S6 and S7, *dsrA* was carried only by Betaproteobacteria. About half of the sulfur cycling-related genes in our statistics were highly correlated with salinity, all of these sulfur cycling-related genes were negatively correlated with salinity (Table 4).

**Table 4:** Pearson correlation between sulfur cycling-related genes and chemical properties

		pH	salinity	NH_4_^+^-N	SiO_3_^2−^-Si	NO_2_^−^-N	PO_4_^3−^-P	NO_3_^−^-N
*soxY*	*r*	0.017	-0.436	0.245	-0.194	0.011	-0.232	-0.148
	*P*	0.967	0.281	0.558	0.645	0.98	0.58	0.726
*soxB*	*r*	0.125	-0.823*	0.199	-0.538	0.194	0.144	-0.081
	*P*	0.767	0.012	0.636	0.169	0.645	0.734	0.848
*soxZ*	*r*	0.628	-0.815*	0.331	-0.19	0.635	-0.062	-0.085
	*P*	0.095	0.014	0.423	0.653	0.09	0.884	0.841
*soxX*	*r*	0.183	-0.465	0.47	-0.14	0.158	-0.321	-0.032
	*P*	0.664	0.246	0.24	0.742	0.71	0.439	0.941
*soxA*	*r*	0.175	-0.433	0.342	-0.349	0.345	-0.455	-0.084
	*P*	0.678	0.284	0.407	0.396	0.403	0.257	0.843
*cysD*	*r*	0.005	-0.28	-0.217	-0.158	-0.103	0.804*	0.143
	*P*	0.99	0.501	0.605	0.708	0.809	0.016	0.735
*cysN*	*r*	0.484	-0.708*	0.664	-0.425	0.472	-0.21	0.354
	*P*	0.224	0.049	0.073	0.294	0.237	0.618	0.389
*sat*	*r*	0.052	-0.464	0.418	-0.316	0.084	-0.332	0.017
	*P*	0.902	0.247	0.303	0.445	0.844	0.422	0.968
*aprA*	*r*	0.275	-0.807*	0.261	-0.557	0.394	-0.101	0.036
	*P*	0.51	0.016	0.532	0.152	0.334	0.811	0.932
*aprB*	*r*	0.305	-0.376	0.36	-0.492	0.328	-0.169	0.696
	*P*	0.462	0.358	0.382	0.216	0.427	0.689	0.055
*dsrA*	*r*	0.5	-0.524	0.628	-0.407	0.542	-0.522	0.492
	*P*	0.207	0.183	0.095	0.317	0.166	0.185	0.215
*dsrB*	*r*	0.47	-0.951**	0.174	-0.571	0.543	0.323	0.157
	*P*	0.24	0.001	0.681	0.139	0.165	0.435	0.71

*Correlation is significant at the 0.05 level; **Correlation is significant at the 0.01 level

## Discussion

Bacterial taxa that were strongly correlated with salinity were first screened out and their differences in relative abundance at different sampling sites were discussed. As can be seen from the figure (Fig. 3b), Alphaproteobacteria and Deltaproteobacteria have high abundances in S5. These classes are ubiquitous in the marine environment and contain many marine species with high abundance[25, 26]. The high abundance of Rhodobacteraceae and Pseudoalteromonadaceae observed in S5 due to the rapid response of these families to the input of foreign nutrients caused by glacial meltwater (Fig. 3c), their high abundance may be an indication of coastal surface water disturbance events [27, 28]. Salinity can be an important factor in driving microbial diversity [11, 29–31] and controls global microbial distribution [11].

Our data confirm that certain bacterial groups are strongly correlated with salinity. For instance, a high abundance of Flavobacteriaceae, Vibrionaceae and Rhodobacteraceae are also detected in S5. Flavobacteriaceae is widespread in freshwater and also a dominant family in the Midtre Lovénbreen glacier[32]. The high abundance of Flavobacteriaceae since this bacterium prefers to utilize complex organic matter by directly attaching to algal cells and algal derivative clastic particles [33], while the freshwater of S5 is more conducive to the growth of algae[34], so salinity indirectly affects the abundance of Flavobacteriaceae. In addition, *Flavobacterium* was the most abundant genus in this family in our study, and it has been shown to be significantly more abundant in low salinity seawater than in high salinity seawater[35]. The high abundance of Vibrionaceae can be ascribed to its association with soil microbiota [36], suggesting that Vibrionaceae may be terrigenous microorganisms, which are imported into the fjords by glacial meltwater and are subjected to salinity stress. The Vibrionaceae is not abundant in high salinity areas due to salt stress. This characterization is consistent with the findings of the L. Lidong that the relative abundance of Vibrionaceae is significantly higher in the melt water of the upper glacial low-salinity region[37]. It has been shown that the abundance of Rhodobacteraceae is higher in the coast than in the offshore and that salinity has a significant negative effect on Rhodobacteraceae[38]. In our study, Rhodobacteraceae is significantly more abundant in S5 than in S7, which may be due to the fact that S5 is a coastal area and salinity is diluted by glacial meltwater. Thus, these results confirm the rule that S5 and S7 have different dominant bacteria due to salinity differences.

Salinity changes most significantly at sampling sites with the input of glacial meltwater. Therefore, we believe that the effect of salinity on the abundance of some bacterial groups is significant. Actinobacteria are common degrading bacteria in soil and ocean [39, 40], which were initially thought to be transferred to the marine environment through terrestrial runoff [40]. However, in our figure, the abundance of Actinobacteria in S6 and S7 is higher than that of S5 (Fig. 3a), and the abundant of Acidimicrobiaceae and Microbacteriaceae in S6 and S7 confirm that Actinobacteria are the resident members of marine environment [41–43] and high-salt environment [44] (Fig. 3c). The abundance of Gammaproteobacteria is higher in high salinity S7 (Fig. 3b), which is consistent with previous studies [45]. Systematic evolution of the proteobacteria along salinity gradients shows the effect of salinity on the structure [30], suggesting that salinity is an important factor controlling the composition of the microbial community in Kongsfjorden. The high abundance ratios of Colwelliaceae, Chromatiaceae, and Alteromonadaceae observed in high salinity S7 may be attributed to their sensitivity to salinity (Fig. 3c), proving that they may belong to the marine bacterium [46–48] and they may have "exclude salt" mechanisms such as osmotic balance and prevent dry [49]. In this adaptation, the microorganism synthesizes the corresponding solute to help stabilize the cell membrane structure.

It can be seen that there are great differences in gene functional abundance between S5 and S7. The abundance of most metabolism-related genes in S5 is high, suggesting that the Kongsfjorden estuary is a highly competitive environment where bacteria must exhibit the ability to take advantage of changing nutrient conditions by demonstrating multiple metabolic pathways [13]. Most of the S5 metabolism-related genes are concentrated in energy metabolism, amino acid metabolism and carbohydrate metabolism (Fig. 5). The influx of glacial meltwater in the upper reaches of Kongsfjorden promotes the accumulation of carbon-rich substances, and the conversion of inorganic carbon to organic matter through carbon sequestration is the main functional attribute of the ecosystem. Chemoautotrophs also use inorganic carbon to some extent [50]. Although heterotrophic prokaryotes utilize organic carbon, they can integrate dissolved inorganic carbon through extensive carboxylation reactions [51]. The high abundance of energy metabolism- related genes and carbohydrate-related genes at the S5 site is due to the rich microbial community at the low salinity site, which constitutes an effective carbon sequestration system in the Fjord estuary through autotrophic and heterotrophic mechanisms. Proteobacteria and actinomycetes have previously been reported to play a major role in carbohydrate metabolism and carbon fixation [51]. The enhancement of amino acid metabolism can enable microorganisms to successfully obtain nutrients in the context of intense nutrient competition. For example, increased nutrient acquisition by highly reactive microbial groups in S5 leads to the production of reactive oxygen species through nutrient oxidation, metabolism, and cellular respiration [52] and causes overexpression of cellular responses to oxidative stress, of which glutathione is a major component [53]. But in a high salinity environment S7, halophilic and salt-tolerant microorganisms must maintain their cytoplasm at least isosmotic with their environment to withstand a high salinity environment [54]. The strategy of some of these organisms is to remove as much salt from the cytoplasm as possible and to accumulate organic solutes to provide osmotic equilibrium. There are a variety of compounds that can be used for this purpose, from glycerol and other sugar alcohols to cytidine [55], which leads to a high abundance of genes related to fat metabolism and nucleotide metabolism in high-salt environments.

We believe that salinity has effects on the abundance of genes associated with the nitrogen cycle, as most of the genes associated with the nitrogen cycle were negatively correlated with salinity. The abundance of nitrification-related genes (*amoC*, *hao*, and *nxrB*) and denitrification-related genes (*nirK*, *nirS*, *norB*, *norC*, and *nosZ*) is high at low salinity sites. Previous studies have found that estuarine nitrifiers grow best at 5–10 ppt and will be inhibited if the salinity exceeds 10 ppt [56], which means that nitrification related genes are most abundant at sites with salinity less than 10 ppt. Denitrification, which returns nitrogen to the atmosphere as N_2_O and N_2_, shows a different relationship with salinity. In some estuaries, the abundance of denitrification-related genes is negatively correlated with salinity in the range of 0–36 ppt [57]. The abundance and potential of denitrifying bacteria are associated with low salinity of around 5 ppt [58, 59]. Salinity can affect denitrification by altering the organic substrates necessary for heterotrophic bacteria [58]. Therefore, the high concentration of NO_3_^−^-N at high salinity can be attributed to the low abundance of denitrifying bacteria at high salinity. In addition, the reduction of dissimilated nitrate to ammonium (DNRA) (*nirB*, *nirD*, and *nrfA*) can compete with denitrification for nitrate, we observed higher concentrations of NH_4_^+^ at the lower salinity sites (Table 1), indicating less NH_4_^+^ consumption, which may be due to increased DNRA activity, further counteract the NH_4_^+^ consumption [60]. A recent study also showed that, although anammox may be inhibited at higher salinity, increases in salinity below 15 ppt can stimulate anammox [61]. This may explain our observation that the abundance of anammox-associated genes (*hdh*) is also high when salinity ranges from 5.1 to 15.16 (Fig. 6a).

We suggest that salinity affects the abundance of genes associated with the nitrogen cycle by affecting gene-carrying microbial populations. Sahan and Muyzer [62] showed that salinity was the main factor controlling the distribution of microorganisms associated with the nitrogen cycle. Gammaproteobacteria and Alphaproteobacteria are dominant in most of the nitrogen cycling-related genes in the three sample sites, but Alphaproteobacteria has a slight limitation under high salinity (Fig. 6b), which is consistent with the low abundance of Alphaproteobacteria in high-salinity areas (Fig. 3b).

Fjords are an important part of the global ecosystem and play an important role in the sulfur cycle. Sulfur oxidation (Sox) is usually driven by sulfur-oxidizing bacteria, which oxidize reduced sulfide compounds, including elemental sulfur, sulfides, and thiosulfates [63]. The Sox enzyme system is widely present in known sulfur oxidizing bacteria. In this study, the Sox enzyme system was detected to contain four gene: *SoxAX*, *SoxY*, *SoxZ*, and *SoxB* (Fig. 7a). Sox-related genes are primarily present in Gammaproteobacteria, Betaproteobacteria, and Alphaproteobacteria (Fig. 7b). Yang et al.Yang, Jiang, Dong, Wu, Hou, Zhao, Sun and Lai [63] have found that the response of a given sulfur oxidizing bacteria population to increases of salinity consists of successive changes in community structure but not of gradual adaptation of the sulfur oxidizing bacteria population. Because sulfur oxidizing bacteria belong to various classes of Proteobacteria, the response pattern of sulfur oxidizing bacteria to increases of salinity is consistent with that of proteobacterial classes [63]. Our study shows that Alphaproteobacteria is dominant at low salinity, while Gammaproteobacteria is dominant at high salinity (Fig. 3b). Consistently, in this study, Alphaproteobacterial sulfur oxidizing bacteria with high abundance are detected in S5, while Gammaproteobacterial sulfur oxidizing bacteria with high abundance are detected in S7 (Fig. 7b). Bacterial sulfate reduction has important ecological and geochemical significance in marine high-salt sediments [64]. Notably, besides the carry of the genes related to sulfate reduction by Gammaproteobacteria and Alphaproteobacteria, Flavobacteriia is dominant in the microbial populations belonging to sulfate reduction related genes *cysD* and *cysN* (Fig. 7b). In addition, Flavobacteria plays an important role in sulfate reduction, which is consistent with the research results of Li, Jing, Xia, Cheung, Suzuki and Liu [33].

## Conclusions

We used metagenomic analysis to demonstrate that changes in salinity can affect the relative abundance of some bacterial taxa on the one hand, and also change the relative abundance of functional genes and genes related to the nitrogen and sulfur cycles on the other hand.

### Supplementary information

ESM 1: Supplemental material (XLSX 12.9 KB)

ESM 2: Supplemental material (XLSX 15.7 KB)

ESM 3: Supplemental material (XLSX 14.1 KB)

ESM 4: Supplemental material (XLSX 14.6 KB)

ESM 5: Supplemental material (PDF 223 KB)

## Data Availability

Raw data have been deposited into the NCBI Sequence Read Archive (SRA) database (Accession Number: SRP319744).
