# Supplementary material for: Metagenomic analysis of the effects of salinity on microbial community and functional gene diversity in glacial meltwater estuary, Ny-Alesund, Arctic
Source: Braz J Microbiol. 2024 Apr 22;55(2):1587–99. doi: 10.1007/s42770-024-01298-x (PMC11153410; doi:10.1007/s42770-024-01298-x)
Supplement: Supplementary file 5 — Supplemental material (PDF 223 KB) [file 42770_2024_1298_MOESM5_ESM.pdf]

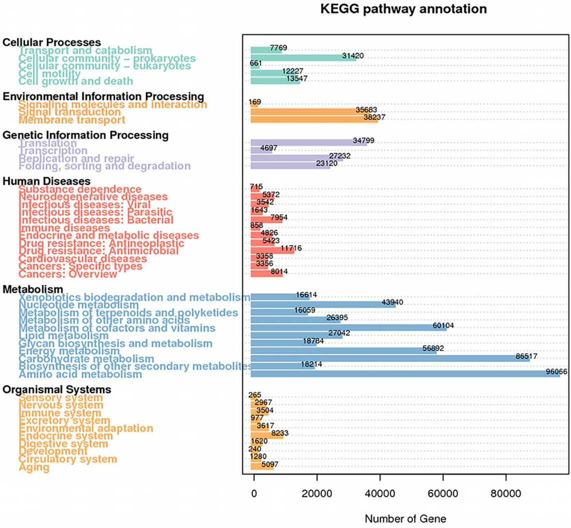

KEGG pathway annotation
Cellular Processes
Transport and catabolism
Cellular community – prokaryotes
Cellular community – eukaryotes
Cell motility
Cell growth and death
Environmental Information Processing
Signaling molecules and interaction
Signal transduction
Membrane transport
Genetic Information Processing
Translation
Transcription
Replication and repair
Folding, sorting and degradation
Human Diseases
Substance dependence
Neurodegenerative diseases
Infectious diseases: Viral
Infectious diseases: Parasitic
Infectious diseases: Bacterial
Immune diseases
Endocrine and metabolic diseases
Drug resistance: Antineoplastic
Drug resistance: Antimicrobial
Cardiovascular diseases
Cancers: Specific types
Cancers: Overview
Metabolism
Xenobiotics biodegradation and metabolism
Nucleotide metabolism
Metabolism of terpenoids and polyketides
Metabolism of other amino acids
Metabolism of cofactors and vitamins
Lipid metabolism
Glycan biosynthesis and metabolism
Energy metabolism
Carbohydrate metabolism
Biosynthesis of other secondary metabolites
Amino acid metabolism
Organismal Systems
Sensory system
Nervous system
Immune system
Excretory system
Environmental adaptation
Endocrine system
Digestive system
Development
Circulatory system
Aging
7769
31420
661
12227
13547
169
35683
38237
34799
4697
27232
23120
715
5372
3542
1643
7954
858
4826
5423
11716
3358
3356
8014
16614
43940
16059
26395
60104
27042
18784
56892
86517
18214
96066
265
2967
3504
977
3617
8233
1620
240
1280
5097
0
20000
40000
60000
80000
Number of Gene
